# Peptide Signatures for Prognostic Markers of Pancreatic Cancer by MALDI Mass Spectrometry Imaging

**DOI:** 10.3390/biology10101033

**Published:** 2021-10-12

**Authors:** Florian N. Loch, Oliver Klein, Katharina Beyer, Frederick Klauschen, Christian Schineis, Johannes C. Lauscher, Georgios A. Margonis, Claudius E. Degro, Wael Rayya, Carsten Kamphues

**Affiliations:** 1 Department of Surgery, Charité—Universitätsmedizin Berlin, Corporate Member of Freie Universität Berlin and Humboldt-Universität zu Berlin, Hindenburgdamm 30, 12203 Berlin, Germany; katharina.beyer2@charite.de (K.B.); christian.schineis@charite.de (C.S.); johannes.lauscher@charite.de (J.C.L.); claudius-erwin.degro@charite.de (C.E.D.); wael.rayya@charite.de (W.R.); Carsten.kamphues@charite.de (C.K.); 2 Berlin Institute of Health, Charité—Universitätsmedizin Berlin, Center for Regenerative Therapies BCRT, Charitéplatz 1, 10117 Berlin, Germany; oliver.klein@charite.de; 3 Institute for Pathology, Charité—Universitätsmedizin Berlin, Corporate Member of Freie Universität Berlin and Humboldt-Universität zu Berlin, Charitéplatz 1, 10117 Berlin, Germany; frederick.klauschen@charite.de; 4 Institute for Pathology, Ludwig-Maximilians-Universität München, 80337 München, Germany; 5 Department of Surgery, Memorial Sloan Kettering Cancer Center, New York, NY 10065, USA; margonig@mskcc.org

**Keywords:** pancreatic cancer, peptide signatures, MALDI-MSI, risk stratification

## Abstract

**Simple Summary:**

Pancreatic cancer remains one of the most lethal tumor entities worldwide given its overall 5-year survival after diagnosis of 9%. Thus, further understanding of molecular changes to improve individual prognostic assessment as well as diagnostic and therapeutic advancement is crucial. The aim of this study was to investigate the feasibility of Matrix-assisted laser desorption/ionization (MALDI) mass spectrometry imaging (MSI) to identify specific peptide signatures linked to established prognostic parameters of pancreatic cancer. In a patient cohort of 18 patients with exocrine pancreatic cancer after tumor resection, MALDI imaging analysis additional to histopathological assessment was performed. Applying this method to tissue sections of the tumors, we were able to identify discriminative peptide signatures corresponding to nine proteins for the prognostic histopathological features lymphatic vessel invasion, lymph node metastasis and angioinvasion. This demonstrates the technical feasibility of MALDI-MSI to identify peptide signatures with prognostic value through the workflows used in this study.

**Abstract:**

Despite the overall poor prognosis of pancreatic cancer there is heterogeneity in clinical courses of tumors not assessed by conventional risk stratification. This yields the need of additional markers for proper assessment of prognosis and multimodal clinical management. We provide a proof of concept study evaluating the feasibility of Matrix-assisted laser desorption/ionization (MALDI) mass spectrometry imaging (MSI) to identify specific peptide signatures linked to prognostic parameters of pancreatic cancer. On 18 patients with exocrine pancreatic cancer after tumor resection, MALDI imaging analysis was performed additional to histopathological assessment. Principal component analysis (PCA) was used to explore discrimination of peptide signatures of prognostic histopathological features and receiver operator characteristic (ROC) to identify which specific *m*/*z* values are the most discriminative between the prognostic subgroups of patients. Out of 557 aligned *m*/*z* values discriminate peptide signatures for the prognostic histopathological features lymphatic vessel invasion (pL, 16 *m*/*z* values, eight proteins), nodal metastasis (pN, two *m*/*z* values, one protein) and angioinvasion (pV, 4 *m*/*z* values, two proteins) were identified. These results yield proof of concept that MALDI-MSI of pancreatic cancer tissue is feasible to identify peptide signatures of prognostic relevance and can augment risk assessment.

## 1. Introduction

Pancreatic cancer was diagnosed in 458,918 patients worldwide in 2018. Despite immense efforts to improve early detection and clinical management, the overall 5-year survival after diagnosis remains 9% [1]. At time of diagnosis the main proportion of patients has advanced stage disease, leaving only 15–20% qualified for potentially curative, resective surgery [2]. Even after successful resection of cancer of the pancreatic head the 5-year survival remains 21% [3]. There is, however, heterogeneity in clinical courses of tumors even within the same stage [4]. This indicates a pressing need to further augment clinical and histopathological staging in categorizing tumor malignancy, behavior and prognosis by additional prognostic markers for proper risk stratification and, consequently, clinical management of exocrine pancreatic cancer. In cases of resectable disease certain subgroups of patients need to be identified that are likely to benefit from neoadjuvant therapy due to aggressive tumor biology or occult metastatic disease. In cases of highly unfavorable tumor biology omitting surgery may be considered to spare hospitalization time at end of life period. In unresectable disease the further prognostic characterization contributes to the decision of the aggressiveness and toxicity of treatment.

Matrix-assisted laser desorption/ionization (MALDI) mass spectrometry imaging (MSI) is an emerging method for molecular analysis on tissue microarrays (TMAs) from obtained biopsies or surgical specimens which preserves the morphological integrity of the analyzed tissue. Therefore, it is enabled to assess the spatial distribution of proteomic analysis and allows further processing and staining of the TMA [5]. Due to its ability of untargeted peptide mapping, corresponding proteins observed do not need to be known in advance and therefore do not require molecule-specific tags [6,7]. Consequently, it allows the spatial correlation of peptide signatures with clinicopathological features. MALDI-MSI can be used to support tissue assessment in large formats and therefore has huge potential for routine clinical application and as pathology aid. A broad range of applications demonstrate that MALDI-MSI is feasible to, e.g., classify tumor subtypes [8,9], predicting therapeutic responses [10] or providing new biological insights into intratumor heterogeneity [9]. It has also been successfully applied to discover prognostic markers for recurrent vs. non-recurrent disease of early-stage high-grade serous ovarian cancer and risk stratification of neuroblastoma [11,12]. As for tissue analysis of pancreatic cancer, MALDI-MSI has so far been applied on pancreatic cryosections of genetically engineered mouse models to differentiate preneoplastic lesions (PanIN, IPMN) from healthy tissue and pancreatic ductal adenocarcinoma (PDAC) as well as to characterize the delivery and distribution of erlotinib in PDAC [13,14].

The aim of this study is to apply this method on formalin-fixed paraffin-embedded tumor tissue of patients with resected PDAC and find peptide signatures correlated to prognostic histopathological characteristics. Thus, to give proof of concept that MALDI-MSI is feasible to identify subgroups of patients with favorable and less favorable tumor biology in patients with PDAC.

## 2. Materials and Methods

### 2.1. Patient Cohort and Histopathological Assessment

In this single center study approved by its local ethics committee, samples of 18 patients with histologically proven exocrine carcinoma of the pancreas that underwent primary oncologic surgery between January 2013 and March 2015 at the Department of Surgery, Campus Benjamin Franklin, Charité—University Medicine Berlin, Germany, were included after informed consent. Demographic and clinicopathological characteristics of the patients are shown in Table 1. Standard protocol of histopathological TNM staging of surgical specimens with additional variables of established prognostic relevance lymphatic vessel invasion (pL), angioinvasion (pV), perineural invasion (P) and histologic grade (Gx-4) was performed for conventional pathological assessment and risk stratification of tumors [15].

### 2.2. Procedure of MALDI-Imaging

TMAs from formalin-fixed, paraffin-embedded tissue from patients diagnosed with exocrine pancreatic cancer were prepared at the Institute of Pathology, Charité—Medical University Berlin. For MALDI imaging, a 6-µm section from a paraffin block was prepared on the microtome and transferred to indium tin oxide slides (Bruker Daltonik, Bremen, Germany) by decreasing concentrations of ethanol (modified after Caprioli et al.) [5] and antigen recovery was performed (modified after Gustafsson et al.) [16]. An automatic spayer was used to apply Trypsin and matrix solutions (α-cyano-4-hydroxycinnamic acid, (HTX Sprayer). In total 550 µL trypsin solution (20 µg, 20 mM ammonium bicarbonate) was applied to the section. After incubating the tissue (2 h at 50 °C; humid chamber), the matrix solution (1 mL 7 g/L α-cyano-4-hydroxycinnamic acid in 50% acetonitrile and 1% trifluoroacetic acid) was applied also with the HTX sprayer (75 °C, estimation cycle 1.80).

### 2.3. MALDI Imaging Analysis

Analyses were performed on 1–7 biologically independent cores of biopsies for each patient group (median 3.5). The tissue cores of the tumor used for analysis contain >80% of tumor cells. MALDI-MSI data acquisition was performed in reflector mode, detection range *m*/*z* 800–3200, 500 laser shots per spot, sampling rate of 1.25 GS/s and grid width of 50 µm on Rapiflex MALDI-TOF using flexControl 3.0 and flexImaging 3.0 (Bruker Daltonik). A peptide calibration standard (Bruker Daltonik) was used for external calibration and spectra processed in flexAnalysis 3.0 (Bruker Daltonik). For the exclusion of possible contaminations such as sodium adducts or peptides, analysis of control areas outside the tissue was performed. Subsequent to the MALDI imaging experiments, the matrix was removed by applying 70% ethanol and the tissue sections were stained with haematoxylin and eosin (H&E) as a histological overview stain [5].

### 2.4. Data Processing

Statistical data was computed using the SCiLS Lab software (Version2021b, SCiLS GmbH, Bremen, Germany). MALDI-MSI raw data was imported into the SCiLS Lab software and then converted to the SCiLS Lab file format. Simultaneously, all data sets were preprocessed to ensure better comparability between the sample sets. Imported data was preprocessed by convolution baseline removal (width: 20) and total ion count (TIC) normalization. Segmentation pipelines were performed for peak-finding and their alignment as published previously [17,18,19]. The orthogonal matching pursuit (OMP) algorithm was used for the selection of peaks and top down segmentations were performed by bisecting k-means clustering, ±0.156 Da interval width, mean interval processing and medium smoothing strength [18,19,20]. As supervised approach receiver operating characteristic (ROC) analyses were performed to detect characteristic peptide values. ROC analysis was used for assessing the quality of all *m*/*z* values within specific ROIs for discrimination between tumor tissues with respective prognostic histopathological features. For this method, the number of spectra in the ROIs of both groups should be approximately the same. Otherwise, 1500 randomly selected spectra per ROI/group were used. To determine statistical significance, discriminating *m*/*z* values (peaks) with an AUC < 0.4 or > 0.6 were subsequently analyzed using the Wilcoxon rank sum test. A *p*-value of <0.001 was assumed as a potential marker. Figures were created using the SCiLS Lab software (Bruker, Bremen, Germany). Supervised principal component analysis (PCA) was conducted to define characteristic peptide signatures differentiating between tumor regions with >80% tumor cell content from groups in terms of absence or presence of prognostic histopathological features. The data was scaled for PCA in a level scaling model using settings to create five components, an interval width of ±0.3 Da, maximal interval processing mode, normalization to total ion count, no noise reduction.

### 2.5. Identification of Peptides by “Bottom-Up”-HPLC Mass Spectrometry

Complementary protein identification was performed on adjacent tissue sections to identify *m*/*z* values by a “bottom-up”-nano liquid chromatography (nLC)-MS/MS approach as published previously [17]. In brief, tissue digestion (20 µg trypsin, 20 mM ammonium bicarbonate/acetonitrile 9:1) was performed via ImagePrep (Bruker Daltonik) followed bypeptide extraction for nUPLC-MS/MS analysis directly from adjacent tissue sections into 40 µL of 0.1% triflouroaceticacid (TFA; 15 min incubation at room temperature). Peptides were separated (60% acetonitrile/in 0.1% formic acid) using an analytical UPLC System (Thermo Dionex Ultimate 3000, Acclaim PepMap RSLC C18 column 75 µm × 15 cm; flow rate 200 nL/min, 70 min) and analyzed via Impact II (QTOF-MS, Bruker Daltonik). All raw spectra from the MS/MS measurement were converted to mascot generic files (.mgf) by the ProteinScape software [21]. Analysis of mass spectra was performed using the Mascot search engine (version 2.4, MatrixScience; UK) searching the UniPort database. The query was performed with the following set of parameters: (i) taxonomy: human; (ii) proteolytic enzyme: trypsin; (iii) peptide tolerance: 10 ppm; (iv) maximum of accepted missed cleavages: 1; (v) peptide charge: 2+, 3+, 4+; (vi) variable modification: oxidation (M); (vii) MS/MS tolerance: 0.8 Da; and (viii) MOWSE score > 25. Identification of MALDI-MSI *m*/*z* values by using an LC-MS/MS reference list requires the accordance of more than one peptide (mass differences < 0.2 Da) to correctly assign the corresponding protein [22]. Peptides with lowest mass difference to the LC-MS/MS reference list value were assumed as a match.

## 3. Results

### 3.1. MALDI-MSI Data and Identification of Discriminative Peptide Signatures for Prognostic Histopathological Tumor Features

**Figure 1:**
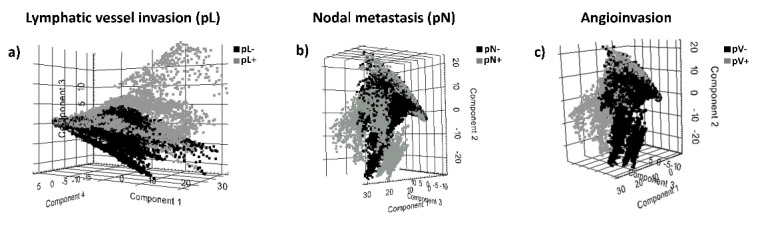
Principal component analysis (PCA) of MALDI-MSI data showing a discrimination of peptide signatures of tumors in terms of absence or presence of the prognostic histopathological features (**a**) lymphatic vessel invasion (pL+ vs. pL−), (**b**) nodal metastasis (pN+ vs. pN) and (**c**) angioinvasion (pV+ vs. pV−).

We evaluated the technical feasibility of MALDI-MSI to identify the peptide signature and potential discriminative peptide signatures of formalin-fixed, paraffin-embedded (FFPE) tissue sections of pancreatic cancer tissue of surgical specimens. In total, 557 aligned *m*/*z* values in the mass range for tryptic peptides (*m*/*z* value range: 800–3200 were extracted from the analyzed tissue sections. In order to identify discriminative peptide signatures linked to prognostic histopathological tumor features (tumor size (pT), nodal metastasis (pN lymphatic vessel invasion (pL), vascular invasion (pV), perineural invasion (P) and histologic grade, Gx-4) principal compound analysis (PCA) was conducted on MALDI-MSI data from the tissue sections. PCA of MALDI-MSI data of tumor regions (>80% tumor cell content) showed a discrimination of peptide signatures of tumors in terms of absence or presence of the prognostic features lymphatic vessel invasion (pL+ vs. pL−), nodal metastasis (pN+ vs. pN−) and angioinvasion (pV+ vs. pV−) (Figure 1). The first principal component explained 54% of the variance. This demonstrates that unsupervised statistical approach results in discriminatory peptide signatures of tumors with lymphatic vessel invasion (pL+ vs. pL−), nodal metastasis (pN+ vs. pN−) and angioinvasion (pV+ vs. pV−) using MALDI-MSI data from pancreatic cancer tissue sections.

**Figure 2:**
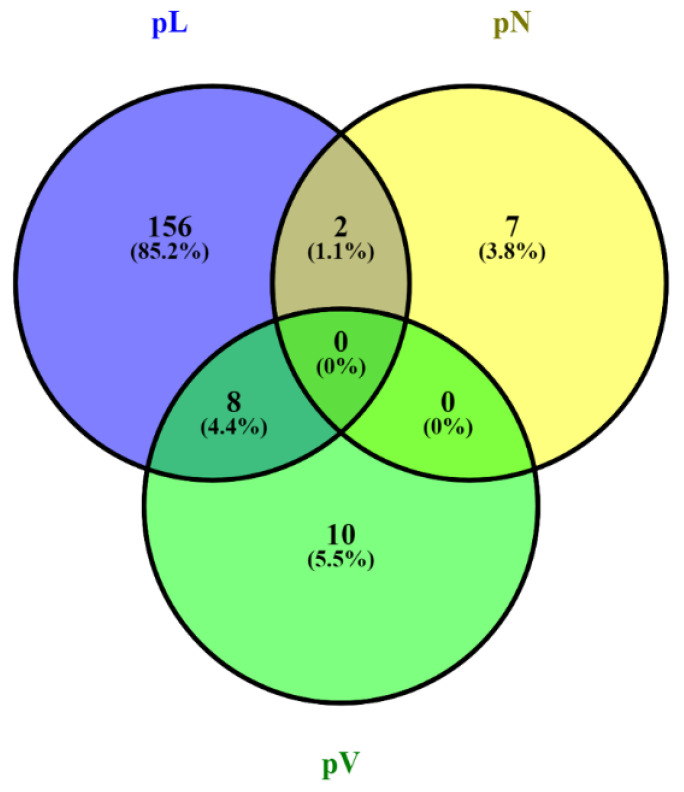
MALDI-IMS derived peptide values discriminative between the prognostic subgroups of lymphatic vessel invasion (pL), nodal metastasis (pN) and angioinvasion (pV) and their overlap. Discriminative peptide values: 156 peptides are unique to distinguish between tumors with lymphatic vessel invasion (pL+) and absence of lymphatic vessel invasion (pL−), seven peptides for nodal metastasis (pN+) and no nodal metastasis (pN−) and ten peptides for angioinvasion (pV+) and absence of angioinvasion (pV−).

In total, MALDI-IMS derived 183 peptide values discriminative between subgroups of patients in terms of the prognostic features lymphatic vessel invasion (pL), nodal metastasis (pN) and angioinvasion (pL) from the 557 aligned *m*/*z* values in the mass range for tryptic peptides in the analyzed tissue sections. The number of unique peptide values among the subgroups of patients with the respective prognostic feature and their overlap is shown in Figure 2.

### 3.2. Identification of Proteins Linked to Discriminative Peptide Signatures from Pancreatic Cancer Tissue Sections

Univariate analysis of MALDI-MSI data has the potential to identify which specific *m*/*z* values are the most discriminative between the prognostic subgroups of patients. Therefore, receiver operator characteristic (ROC) analyses were applied to the total 557 aligned *m*/*z* peaks from tumor cell-rich areas in paired comparison of tissue sections of tumors with lymphatic vessel invasion (pL+) and absence of lymphatic vessel invasion (pL−), nodal metastasis (pN+) and without nodal metastasis (pN−) and angioinvasion (pV+) or absence of angioinvasion (pV−). Consequently, to identify the proteins corresponding to the discriminatory tryptic peptide fragments, we used a bottom-up nanoliquid chromatography-tandem mass spectrometry (nanoLC-MS/MS) approach in adjacent tissue sections. This analysis assigned 154 of the 557 *m*/*z* values to peptides corresponding to proteins identified by nanoLC-MS/MS. Corresponding proteins to *m*/*z* values are correctly identified when the validating approach (nanoLC-MS/MS in this case) identifies at least two peptides (detected in MALDI-MSI) from the same protein, whose spatial differential intensities are similar. This requisite was met for eight proteins (16 *m*/*z* values), one protein (2 *m*/*z* values) and two proteins (4 *m*/*z* values) for the respective prognostic feature lymphatic vessel invasion (pL), nodal metastasis (pN) and angioinvasion (pV). The corresponding proteins show increased intensity distribution in the subgroup of patients with the prognostic characteristic of lymphatic vessel invasion (pL+, AUC > 0.6, *p* < 0.001) and angioinvasion (pV+; AUC > 0.6, *p*< 0.001). In contrast, the corresponding protein shows decreased intensity distribution in the subgroup of patients with nodal metastasis (pN+, AUC < 0.4, *p* < 0.001) (see Table 2).

Specifically, in pancreatic cancer tissue sections, the explored peptide signature (MALDI imaging derived discriminative peptide values) for lymph angioinvasion (16 *m*/*z* values, pL) corresponds to the eight identified proteins Actin, cytoplasmic (1198 Da, 1790 Da), Collagen alpha-2(I) chain (1547 Da, 1562 Da), Collagen alpha-3(VI) chain (805 Da, 1467 Da), Filamin-B (1628 Da, 1766 Da), Histone H1.3 (1326 Da, 2059 Da), Spectrin beta chain, non-erythrocytic 1 (958 Da, 2059 Da), Valosin-containing protein (1690 Da, 1777 Da) and Vinculin (1269 Da, 1428 Da). Peptides of these corresponding proteins show increased intensity distribution in the subgroup of patients with the prognostic feature lymphatic vessel invasion (pL+, AUC > 0.6, *p* < 0.001) in contrast to patients with absence of lymphatic invasion (pL−). The peptide signature for angioinvasion (4 *m*/*z* values, pV) corresponds to the proteins Collagen alpha-2(I) chain (1562 Da, 2026 Da) and Myosin-11 (2056 Da, 2706 Da). Peptides of both corresponding proteins show increased intensity distribution in the subgroup of patients with the prognostic feature angioinvasion (pV+, AUC > 0.6, *p* < 0.001) in contrast to patients with absence of angioinvasion (pV−). The peptide signature for nodal metastasis (2 *m*/*z* values, pN) corresponds to the protein Histone H1.3 (831 Da, 1326 Da). The corresponding peptides of Histone H1.3 show decreased intensity distribution in the subgroup of patients with the prognostic feature nodal metastasis (pN+, AUC < 0.4, *p* < 0.001) in contrast to patients with absence of nodal metastasis (pN−) (see Table 2).

**Figure 3:**
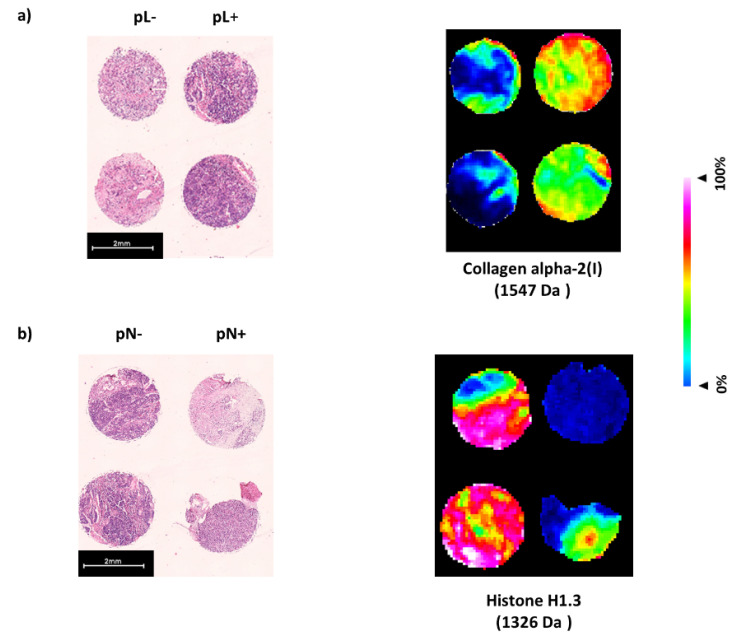
Differential spatial distribution and intensity of the subgroups with and without the respective prognostic histopathological feature (**a**) lymphatic vessel invasion, pL, (**b**) nodal metastasis, pN) for corresponding proteins. Peptide (1541 Da) from Collagen alpha-2(I) shows increased intensity distribution in patients with lymphatic vessel invasion (pL+) whereas peptide (1326 Da) from Histone H1.3 shows decreased intensity distribution in patients with nodal metastasis (pN+). For orientation hematoxylin and eosin stained tissue sections are show on the left.

A depiction of the spatial distribution and spatial intensity of two selected peaks of discriminative proteins for the respective prognostic feature is shown in Figure 3.

## 4. Discussion

The prognosis of exocrine pancreatic cancer is generally poor. Only about 20% of patients qualify for curative, resective surgery at time of diagnosis [2]. Even after successful resection the survival rate remains on an unsatisfactory level of 21% leaving a survival rate of 9% of all patients with the disease after 5 years [3]. The therapeutic classification at diagnosis reaches from resectable to borderline-resectable to non-resectable, palliative disease.

Tumors are considered resectable in absence of infiltration of the coeliac trunk, the superior mesenteric artery and distant metastasis (other organs, peritoneal, distant lymph nodes). Yet, within the group of tumors considered resectable there is a large prognostic heterogeneity even within the same stage (IIa 16.5% to 36.8%, *p* < 0.002; IIb 0% to 59.8%, *p* < 0.001) [4]. This indicates a lack of understanding which patients after upfront tumor resection have favorable or unfavorable tumor biology. In clinical management, surgical resection of the tumor can fail in patients with biologically aggressive disease that do not benefit from extensive, high-morbidity resection at end-of-life period. Apart from the potential of increasing the resectability rate of pancreatic cancer in cases of borderline-resectability by neoadjuvant therapy, preoperative treatment is emerging for primarily resectable disease with the potential to improve prognosis [23]. In this context, exact understanding of tumor biology and risk stratification is crucial for deciding what patients may profit and which need to be precluded because of probable presence of more advanced disease and, consequently, exclusion from curative, surgical therapy after preoperative treatment. In non-resectable cases exact assessment of prognosis can contribute to the choice of treatment regime in terms of toxicity to provide maximum life quality (e.g., FOLFORINOX vs. Gemcitabin-based).

In the performed analysis of this study, specific peptides linked to a signature of proteins for the prognostic histopathological characteristics lymphatic vessel invasion (pL), nodal metastasis (pN) and angioinvasion (pV) were found by MALDI-MSI. Therefore, we present a proof of concept for the technical feasibility of MALDI-MSI to describe prognostically relevant peptide signatures for the further risk stratification of pancreatic cancer beyond standard histopathological assessment and staging.

Additional to this general feasibility of MALDI-MSI, the identified proteins and their prognostic relevance were reviewed according to their concordance to pre-existing literature. All of the encountered peptides and correlated proteins were significantly associated with the respective histopathological characteristic when an increased intensity distribution was seen (AUC > 0.6, *p* < 0.001) except for a decreased intensity distribution of Histone H1.3 in tumors with nodal metastasis (pN+). In consideration of the fact that the exact prognostic role of the majority of these identified proteins is not yet fully resolved, in concordance to our findings Actin, cytoplasmic 1, Collagen alpha-2(I) chain, Collagen alpha-3(VI), Filamin-B and Myosin-11 have been associated with poor prognosis in pre-existing studies using different methods of molecular analysis such as Real-time PCR, western blotting, liquid chromatography-mass spectrometry and immunohistochemical staining [24,25,26,27] Furthermore, Valosin-containing protein (VCP) is known to be a prognosticator for poor prognosis in pancreatic cancer as well as other tumor entities and VCP inhibitors are currently being researched as potential therapeutic target for cancer therapy [28,29].

Noticeably, a large portion of the identified peptides is correlated to proteins that are part of the extracellular matrix (ECM). Generally, MALDI imaging experiments mainly address structural proteins, such as those located in the ECM, since methodically an enzymatic digestion of the surface of TMAs is performed. However, although stromal cells produce over 90% of the ECM mass [30], it was demonstrated that proteins of the ECM are highly expressed in pancreatic cancer cells [31] and elevated levels of ECM proteins derived from tumor cells of pancreatic cancer, but not those produced exclusively by stromal cells, tend to correlate with poor patient survival in pancreatic cancer [30]. The identified proteins are involved in cell migration (Collagen alpha-2(I) chain) [32], effected cancer cell motility (Actin) [33], promotion of metastasis (Valosin-containing protein) [34], cancer cell viability, angiogenesis (Collagen alpha-3(VI) chain) [35] and tumor progression (Collagen alpha-2(I) chain) [36].

In total, MALDI-MSI was able to successfully identify peptide signatures corresponding to altered intensity distribution of nine proteins from 18 patients with exocrine pancreatic cancer that were significantly correlated with poor prognostic parameters lymphatic vessel invasion (pL), nodal metastasis (pN) and angioinvasion (pV, *p* < 0.001). MALDI-MSI is an innovative technology in assisting risk classification taking into account tumor heterogeneity based on spatial peptide signatures [37]. In the presented pilot study, we could demonstrate that MALDI-MSI is feasible to identify peptide signatures of prognostic relevance and can augment risk assessment in pancreatic cancer. In subsequent large-scale studies, these peptide signatures in combination with, e.g., machine learning algorithms [8], leave-one-out cross-validation methods [38] or linear discriminant analysis [39] and deep learning approaches or convolutional neural network (CNN) [40] have to verify to these observations. Multiplex immunohistochemistry (IHC) as well as imaging Cytometry by time of flight (CyTOF) technologies [41] enable the spatial protein target analysis in tissue sections and, thus, represent capable tools to verify identified protein alterations from MALDI-MSI studies. In order to verify protein findings in large-scale cohorts a highly multiplexed IHC MALDI-MSI approach (up to 12-plex) is promising to confirm proteins from MALDI-MSI findings as parallel untargeted label-free investigation of small molecules [42].

The used workflows in this study are applicable to the analysis of larger sample cohorts. Thus, this work represents an important requisite for larger studies for intensified risk assessment of pancreatic cancer using MALDI-MSI.

## 5. Conclusions

Pancreatic cancer has a poor overall prognosis with tumor heterogeneity that is not sufficiently assessed by current conventional risk assessment. In the context of this poor prognosis, high-morbidity resection and toxicity of conservative treatment options an advancement of risk stratification is crucial. In conclusion, this proof of concept study showed the feasibility of MALDI-MSI to identify peptide signatures corresponding to prognostic features of pancreatic cancer.

## Figures and Tables

**Table 1:** Demographic and clinicopathological characteristics of patient cohort.

Patients	*n* = 18
Age	
median age (years)	67
age range (years)	36–77
Sex	
Female	8 (44%)
Male	10 (56%)
Location of main tumor mass	
Pancreatic head	14 (77%)
Pancreatic body	1 (6%)
Pancreatic tail	3 (17%)
Histopathological characteristics	
pT1	1 (6%)
pT2	1 (6%)
pT3	16 (88%)
pN+	12 (67%)
pN−	6 (33%)
G1	1 (6%)
G2	11 (61%)
G3	5 (27%)
G4	1 (6%)
PN+	11 (61%)
pL+	8 (44%)
pL−	10 (56%)
pV+	5 (27%)
pV−	13 (73%)
Adenocarcinoma	17 (94%)
Acinar cell carcinoma	1 (6%)

**Table 2:** Differential intensity distributions of peptides (MALDI-MSI) and their corresponding proteins in tissue sections from pancreatic cancer.

MSI Mr [*m*/*z*] [Da]	Lymphatic Vessel Invasion (pL+) vs. None (pL−) (AUC)	LC-MS Mr [Da]	Deviation Δ [Da]	Protein
1198.839	0.6005	1198.7052	0.1338	Actin, cytoplasmic 1
1790.828	0.6128	1790.8874	−0.0594	
1547.791	0.6213	1547.7901	0.0009	Collagen alpha-2(I) chain
1562.794	0.6343	1562.7900	0.0040	
805.481	0.6001	805.4568	0.0242	Collagen alpha-3(VI) chain
1467.68	0.6005	1467.7243	−0.0443	
1628.804	0.6099	1628.8466	−0.0426	Filamin-B
1766.824	0.6078	1766.9417	−0.1177	
1326.808	0.6125	1326.7631	0.0449	Histone H1.3
2059.968	0.6056	2060.1222	−0.1542	
958.504	0.6056	958.5309	−0.0269	Spectrin beta chain, non-erythrocytic 1
2059.068	0.6165	2059.1005	−0.0325	
1690.913	0.6006	1690.8475	0.0655	Valosin-containing protein (VCP)
1777.926	0.6156	1777.9513	−0.0253	
1269.65	0.6235	1269.6794	−0.0294	Vinculin
1428.674	0.6260	1428.7041	−0.0301	
831.585	0,3786	831.4925	0.0925	Histone H1.3
1326.808	0,3985	1326.7631	0.0449	
1562.794	0.60311	1562.7900	0.0040	Collagen alpha-2(I) chain
2026.963	0.6018	2027.0120	−0.0490	
2056.067	0.6331	2056.0459	0.0211	Myosin-11
2706.264	0.6078	2706.2320	0.0320	

## Data Availability

The data presented in this study are available on request from the corresponding author. The data are not publicly available due to privacy.
